# Electroactive Microbiomes: Electron Transfer Mechanisms and Environmental Biotechnology Applications

**DOI:** 10.1111/1758-2229.70419

**Published:** 2026-09-22

**Authors:** Timoth Mkilima

**Affiliations:** ^1^ Department of Environmental Engineering and Management The University of Dodoma Dodoma Tanzania

**Keywords:** bioelectrochemical systems, electroactive biofilms, electroactive microbiomes, environmental microbiology, extracellular electron transfer

## Abstract

Electroactive microbiomes are mixed microbial communities in which one or more members exchange electrons with extracellular minerals, redox‐active compounds, electrodes, or partner organisms and in which community interactions materially influence net electron flow. This review develops a mechanism–interface–function–readiness framework that connects extracellular electron transfer (EET) with biofilm ecology, microbiome–electrode organisation, environmental process performance, and translational maturity. Direct transfer through multiheme cytochromes and conductive structures, mediated transfer through soluble redox shuttles, and interspecies electron transfer are evaluated as system‐dependent pathways rather than universally ranked mechanisms. Particular emphasis is placed on wastewater treatment and resource recovery, anaerobic digestion, pollutant and metal transformation, soil and sediment bioelectrochemistry, carbon conversion, microbial fuel cells, microbial electrolysis cells, electro‐fermentation, and microbial electrosynthesis. Multi‐omics, metabolic modelling, synthetic biology, advanced materials, and artificial intelligence are examined according to the strength of their direct evidence in electroactive systems. Across applications, performance depends strongly on reactor configuration, electrode properties, inoculum, biofilm architecture, mass and charge transport, substrate loading, and normalisation basis, making unqualified cross‐study numerical comparison inappropriate. Major barriers are long‐term stability, mechanistic attribution in mixed communities, standardisation, scale‐up, energy and mass balances, biosafety, techno‐economic feasibility, and regulatory compatibility.

## Graphical abstract

Electroactive microbiomes couple direct, mediated, conductive, and interspecies electron transfer with biofilm organisation to drive wastewater treatment, bioremediation, resource recovery, carbon conversion, and biosensing. Their performance depends on balancing electron transfer, mass transport, community structure, and electrode–biofilm interactions under realistic environmental conditions.

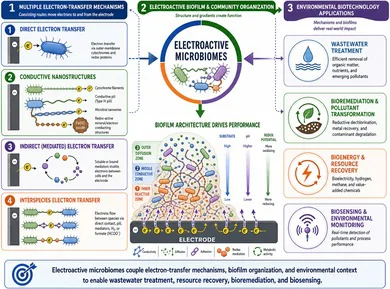

## 1 Introduction

The search for sustainable and carbon‐neutral biotechnology has intensified as societies seek cleaner ways to produce energy, recover resources, treat waste, and reduce environmental pollution. Many conventional technologies still depend on fossil energy, chemical inputs, and linear treatment models in which waste is removed rather than converted into value. Microbial systems offer a different route because microorganisms can transform organic matter, recycle nutrients, drive redox reactions, and adapt to diverse environments (Zhou, Li, et al. 2023; Jatoi et al. 2026). Among these biological processes, microbial electron flow is especially important. It controls how microorganisms gain energy, interact with minerals and chemicals, degrade pollutants, and participate in carbon, nitrogen, sulphur, and metal cycles (Lu et al. 2025). This has created growing interest in engineering microbial communities as living platforms for energy conversion, environmental remediation, industrial production, and bioelectronic sensing.

Electroactive microorganisms were first recognised through their ability to exchange electrons with extracellular insoluble materials, minerals, or electrodes (Conners et al. 2022; Digel et al. 2024). Here, extracellular electron transfer (EET) refers to the movement of reducing equivalents across the cell envelope to or from extracellular electron donors or acceptors. Early work focused mainly on individual current‐producing bacteria, but the field has expanded from isolated strains to electroactive microbiomes: mixed communities in which at least one member performs EET and community interactions, spatial organisation, or metabolic coupling materially affect net electron flow and system function (Zhao et al. 2021). This community‐level definition is important because natural and engineered environments rarely depend on one organism alone. Electron flow can emerge from cooperation among bacteria, archaea, fungi, and other community members, and electroactive microbiomes are now investigated in soils, sediments, wetlands, wastewater systems, anaerobic digesters, industrial bioreactors, and selected host‐associated or bioelectronic settings (Garbini et al. 2023). Their environmental significance lies in coupling microbial metabolism to mineral redox cycling, pollutant transformation, carbon turnover, syntrophic metabolism, and engineered electron recovery.

The function of electroactive microbiomes depends on several molecular and structural mechanisms. Some microorganisms transfer electrons directly through outer‐surface proteins, especially multiheme cytochromes, or through conductive pili and microbial nanowires that extend electron flow beyond the cell surface (Petersen et al. 2026; Edwards et al. 2020). Others rely on indirect transfer, in which soluble redox‐active compounds such as flavins, quinones, phenazines, or humic substances carry electrons between cells and external acceptors (Liu et al. 2018; Hazzan et al. 2023). Electron transfer can also occur between different microbial species, allowing syntrophic partners to share reducing power during anaerobic metabolism (Khalid et al. 2025). Biofilms are central to these processes because they bring cells, extracellular polymers, conductive structures, metabolites, and surfaces into close contact. A well‐organised electroactive biofilm can improve electron transfer, but excessive thickness, poor conductivity, or limited diffusion can reduce performance. Thus, electroactivity is not only a property of a microbe (Conners et al. 2022). It is shaped by community structure, biofilm architecture, redox chemistry, and the surrounding material interface.

These mechanisms support applications across several sectors, but the evidentiary maturity is uneven. Environmental microbiology provides the central application domain: electroactive communities participate in wastewater treatment, pollutant transformation, soil and sediment redox processes, resource recovery, and carbon conversion (Garbini et al. 2023). Broader biological‐remediation evidence provides additional context for metal transformation and immobilisation in contaminated soils (Zheng et al. 2024), while electroactive‐microbiome‐specific metal claims are evaluated separately according to direct mechanistic evidence. Energy and industrial biotechnology extend the same environmental electron‐flow principles through microbial fuel cells, microbial electrolysis cells, electro‐fermentation, microbial electrosynthesis, and waste‐to‐chemical processes (Boto et al. 2025; Gong et al. 2020). Microbial electrochemical biosensing provides direct but narrower evidence (Bindu et al. 2024), whereas host‐associated and bioelectronic concepts are treated as translational boundary areas rather than established environmental‐microbiome applications. Advances in materials science, synthetic biology, multi‐omics, metabolic modelling, and artificial intelligence can improve electrode design, functional attribution, community engineering, and process prediction, but their contribution is strongest when predictions are tied to electrochemical and microbiological validation (Roy et al. 2026).

The literature is fragmented across microbiology, electrochemistry, environmental science, materials engineering, and industrial biotechnology, and recent reviews already cover individual EET mechanisms, electroactive organisms, or bioelectrochemical‐system enhancement strategies (Thapa et al. 2022; Wang et al. 2026). The distinct contribution of this review is therefore not another catalogue of organisms and applications. It uses four linked analytical layers: molecular electron‐transfer mechanism, community and biofilm–electrode organisation, environmental process function, and evidence‐based translational readiness. Within this structure, mechanistic claims are separated from reactor‐level associations, conflicting findings are compared explicitly, quantitative results are interpreted within their experimental context, and mature environmental applications are distinguished from exploratory extensions. The review consequently connects biological mechanism with environmental process design while identifying where evidence is direct, where it is system‐specific, and where further validation is required before scale‐up or cross‐sector translation.

## 2 Methodology

This review used a structured critical narrative synthesis focused on electroactive microbiomes and related bioelectrochemical systems. Searches covered Scopus, Web of Science, PubMed, and Google Scholar and were iteratively updated, with final literature coverage through 19 August 2026. The core concept block was (‘electroactive microbiome*’ OR ‘electroactive microorganism*’ OR exoelectrogen* OR electrotroph*) AND (‘extracellular electron transfer’ OR EET OR ‘direct electron transfer’ OR ‘mediated electron transfer’ OR ‘microbial nanowire*’ OR ‘conductive pili’ OR ‘*c*‐type cytochrome*’ OR ‘interspecies electron transfer’). Environmental searches combined the core terms with wastewater OR bioremediation OR sediment* OR soil* OR ‘anaerobic digestion’ OR methane OR ‘heavy metal*’ OR pollutant* OR ‘resource recovery’ OR ‘carbon dioxide’ OR CO2. Engineering searches added ‘bioelectrochemical system*’, ‘microbial fuel cell*’, ‘microbial electrolysis cell*’, ‘microbial electrosynthesis’, ‘electro‐fermentation’, biofilm*, or electrode*. Multi‐omics and design searches added metagenom*, metatranscriptom*, proteom*, ‘metabolic model*’, ‘synthetic biology’, ‘machine learning’, or ‘artificial intelligence’. Boundary searches combined the core terms with biosensor*, ‘host‐associated’, gut, bioelectronic*, or ‘living material*’. Search syntax was adapted to the operators supported by each database while preserving these concept blocks.

Records were deduplicated and screened first by title and abstract and then by full text for mechanistic relevance, environmental relevance, or direct contribution to electroactive‐system design. Eligible sources were peer‐reviewed studies that examined EET‐capable microorganisms or consortia, electroactive biofilms, microbe‐electrode interactions, interspecies electron transfer, bioelectrochemical reactor performance, or environmental applications involving wastewater, anaerobic digestion, pollutant and metal transformation, soils, sediments, resource recovery, or carbon conversion. Foundational older studies were retained when they established mechanisms, model organisms, or reactor concepts still required to interpret current evidence. Primary experimental studies were prioritised for claims about nanowire structure, conductivity, direct interspecies electron transfer (DIET), electron uptake, biofilm thickness, and quantitative process performance; reviews were used primarily for broader background and synthesis. Purely abiotic electrochemistry, materials studies without a microbial functional link, and biomedical or electronic studies without a direct electroactive‐microbiome connection were excluded from mechanistic inference and retained only where they defined a clearly labelled translational boundary. Because the objective was a structured critical synthesis rather than a systematic review or statistical meta‐analysis, screening was evidence‐oriented and no PRISMA‐style record‐flow count is presented; the synthesis instead reports the databases, search concept blocks, eligibility logic, evidence hierarchy, and quantitative‐comparison rules used to determine inferential weight.

The synthesis evaluated evidence across four dimensions: mechanism, microbiome–interface organisation, environmental or process function, and translational readiness. Evidence was graded qualitatively according to inferential proximity. The highest‐confidence tier comprised primary studies with direct mechanistic intervention or convergent structural, electrochemical, molecular, or genetic evidence. A second tier comprised reactor or mixed‐community studies demonstrating process‐level responses in electroactive systems but with incomplete pathway attribution. Reviews and cross‐study syntheses were used to establish field‐level patterns rather than to prove specific mechanisms. Evidence extrapolated from adjacent biotechnology, materials, or biomedical fields was treated as hypothesis‐generating only. Conflicting findings were retained and compared when differences could plausibly arise from organism, mediator chemistry, electrode material or potential, biofilm architecture, reactor configuration, substrate, or operating conditions.

Quantitative values were interpreted only with their experimental context, including reactor configuration, electrode area and material, inoculum or strain, substrate and loading, hydraulic retention time where applicable, applied potential or external resistance, biofilm state, operation duration, and the basis used to normalise current, power, removal, or product formation. Values reported on incompatible area, volume, mass, or time bases were not treated as direct benchmarks. The review is therefore a structured critical synthesis rather than a statistical meta‐analysis. Its purpose is to identify reproducible mechanisms, explain system‐dependent differences, compare application maturity, and distinguish robust environmental evidence from preliminary or extrapolative directions.

## 3 Fundamentals of Electroactive Microbiomes

### 3.1 Defining the Electroactive Microbiome: The Problem of Binary Classification

The classification of microorganisms as either electroactive or non‐electroactive has been a necessary but potentially limiting framework inherited from early research on pure cultures. This binary approach emerged because extracellular electron transfer could initially only be confirmed through direct electrochemical measurements of isolates in bioelectrochemical systems, a method that systematically excludes organisms that require syntrophic partnerships or specific spatial configurations to exchange electrons with electrodes. The utility of this framework has been substantial. It enabled the identification of *Geobacter sulfurreducens* and *Shewanella oneidensis* as model electroactive organisms, providing foundational knowledge about the molecular components of electron transfer pathways, including multiheme cytochromes and conductive pili (Hernandez and Osma 2020). The binary classification also supports experimental reproducibility, as researchers can reliably predict whether a pure culture will generate current under standard conditions.

However, this dichotomous framework has several structural limitations when extended to complex microbiomes. As noted by Korth and Harnisch (2026), electroactive microorganisms are defined by their ability to perform extracellular electron transfer, which involves extending the electron transfer chain across insulating membranes to exchange electrons with conductive structures in their surroundings. Yet this definition becomes ambiguous when multiple species coexist, because community‐level electroactivity can emerge from interactions that do not require every member to possess canonical electron transfer machinery. Hernandez and Osma (2020) highlighted a related conceptual bottleneck: the increasing diversity of characterised microorganisms poses an enormous challenge, and a definitive model of electroactivity remains to be established. Furthermore, the binary framework cannot easily accommodate the distinction between primary electroactive microorganisms, which establish direct faradaic communication with electrodes, and secondary electroactive systems, where electron exchange occurs through diffusible redox mediators or modification of environmental conditions.

The field is therefore moving towards a context‐dependent view of electroactivity rather than treating it as an intrinsic binary property of isolated cells. Korth and Harnisch (2026) describe microorganisms as living catalysts whose biological traits and energy requirements interact with electrochemical operating conditions. This has an important design consequence: direct‐contact and mediated transfer should not be ranked as universally faster or better pathways. Direct transfer can reduce dependence on diffusible shuttles and their loss or degradation, whereas mediated transfer can extend electron exchange to cells that are spatially separated from the electrode. Relative electron‐transfer rates depend on organism, mediator identity and concentration, electrode material and potential, biofilm architecture, mass transport, substrate supply, and hydrodynamic conditions. Predictive electroactive‐microbiome engineering therefore requires system‐specific identification of the dominant transfer route and its limiting step rather than a general assumption that one mechanism intrinsically outperforms the other.

### 3.2 Taxonomic Diversity: Between Overgeneralisation and Excessive Specificity

A persistent challenge in the literature concerns how broadly to generalise electroactive capabilities across the tree of life. The problem is that most electroactive organisms resist laboratory isolation, meaning culture‐dependent surveys systematically underestimate true phylogenetic diversity (Temirbekova et al. 2023; Lovley and Holmes 2022). The utility of focusing on model genera such as *Geobacter* and *Shewanella* has been demonstrated repeatedly. These organisms have enabled detailed mechanistic studies of electron transfer, including the roles of multiheme cytochromes, quinones, and flavins, and they remain the reference points against which newly discovered electroactive organisms are compared (Hu et al. 2021; Mishra et al. 2026).

Reliance on model organisms also obscures the environmental diversity and context dependence of electroactivity. In a primary survey of 160 samples from eight rivers in the Pearl River Delta, Yang, Dong, et al. (2024) found that pollution status, water quality, and sediment depth were associated with distinct electroactive‐microorganism assemblages, demonstrating that environmental filtering can restructure candidate EET‐capable communities at field scale. The phylogenetic breadth of electroactivity is further illustrated by cable bacteria, filamentous members of the Desulfobulbaceae that conduct electrons over centimetre scales. Ley et al. (2024) compiled 1876 long 16S rRNA gene sequences from 92 aquatic locations and identified 90 potential species‐level clades within six genus‐level clusters, indicating substantially greater diversity than the formally named cable‐bacterium taxa. Their distribution across salinity regimes, sulphide conditions, and deep‐sea settings further shows that taxonomic occurrence alone does not define function; electroactivity must be evaluated together with environmental conditions and direct functional evidence (Ley et al. 2024).

The structural limitation that emerges from these observations is that phylogenetic markers alone cannot reliably predict electroactive function. Genome‐scale and environmental metagenomic studies have shown that phylogenetic trees based on marker genes such as 16S rRNA do not reliably predict metabolic traits, and that functional characteristics can be strongly decoupled from single‐gene phylogenies (Parks et al. 2018). A similar challenge applies to electroactive microbiomes: two organisms sharing high 16S rRNA sequence identity may differ completely in their electron transfer capabilities due to the presence or absence of mobile genetic elements encoding cytochromes or pilins, while distantly related organisms may converge on similar electroactive phenotypes through different molecular mechanisms. The development of functional trait databases, such as BactoTraits, which provides 19 traits for 19,455 bacterial strains, including oxygen preference, cell size and shape, motility, and pH and temperature optima (Cébron et al. 2021), offers a potential path forward. Adapting such trait‐based frameworks to specifically capture determinants of extracellular electron transfer could reduce reliance on phylogenetic proxies and improve predictive capabilities for electroactive microbiome engineering.

### 3.3 Ecological Distribution: Environmental Filters and Inoculum Source Effects

The observation that electroactive microorganisms appear in diverse habitats has motivated a standard practice in bioelectrochemical system development: enriching electroactive communities from locally available inocula, including sediments, soils, wastewater, and even animal guts, with the expectation that electroactive populations will reliably establish themselves under appropriate electrochemical conditions. This approach has proven useful in practice, as the current generation has been reported from inocula sourced from freshwater lakes, marine sediments, salt marshes, paddy soils, and constructed wetlands (Srivastava et al. 2020). The underlying assumption is that electroactive capacity is sufficiently widespread that enrichment will succeed regardless of inoculum origin, given suitable electrode potential and substrate availability.

However, this assumption obscures important variability in the environmental drivers that shape electroactive community assembly. Electroactive microorganisms have been detected in a wide range of habitats, including freshwater and marine sediments, soils, wetlands, wastewater systems, and animal‐associated environments, suggesting that extracellular electron transfer is a widespread ecological strategy rather than a niche metabolic trait (Lovley and Holmes 2022). Despite the extraordinary microbial diversity of soils, the ecology and distribution of electroactive populations in terrestrial environments remain comparatively understudied relative to aquatic sediments (Philippot et al. 2024). Environmental selection for electroactive microorganisms is influenced by the availability of insoluble electron acceptors such as Fe(III) and Mn(IV) oxides, the abundance of competing soluble electron acceptors including oxygen and nitrate, and the presence of conductive minerals that can facilitate extracellular electron transfer (Light et al. 2019). Redox‐stratified and oxygen‐limited environments frequently promote extracellular respiration because the reduction of solid‐phase electron acceptors can provide a favourable alternative to fermentative metabolism under anaerobic conditions (Ciemniecki and Newman 2020). Conversely, environments rich in soluble electron acceptors are generally expected to support lower relative abundances of electroactive microorganisms. However, systematic comparisons across environmental gradients remain limited, constraining efforts to identify the environmental factors that most strongly govern electroactive community assembly.

The practical implication for bioelectrochemical system design is that inoculum source effects are likely substantial but poorly characterised. Tian et al. (2024) provided insight into assembly mechanisms in constructed wetland systems, finding that deterministic processes rather than stochastic assembly dominated microbial community structure, with dissolved oxygen and inorganic nitrogen emerging as the principal environmental factors influencing community dynamics. Extending such assembly mechanism analyses to electroactive microbiomes could help predict which inoculum sources and operating conditions reliably produce high‐performing communities. Until predictive frameworks are available, reactor development will likely remain empirical, requiring case‐by‐case optimisation for each new application and inoculum source.

### 3.4 Evolutionary Significance: Adaptive Value Versus Ancestral Constraint

A fundamental unresolved question concerns whether electroactivity confers a specific fitness advantage that explains its phylogenetic distribution, or whether it represents an ancestral metabolic capability that has been selectively lost in lineages that colonised environments with soluble electron acceptors. The selective advantage argument is supported by thermodynamic reasoning. In anoxic environments lacking soluble electron acceptors, organisms capable of transferring electrons to insoluble Fe(III) or Mn(IV) oxides can respire rather than ferment, yielding substantially more energy per mole of organic carbon oxidised. This advantage has been documented in natural habitats such as mangrove sediments, where electroactive bacteria completely oxidised organic matter to carbon dioxide using Mn(IV) and Fe(III) as electron acceptors (Garbini et al. 2023). Direct interspecies electron transfer between syntrophic partners also offers advantages over hydrogen‐mediated syntrophy, supporting faster conversion rates and more thermodynamically favourable cooperation.

An ancestral origin of EET remains a testable evolutionary hypothesis rather than an established conclusion. Korth and Harnisch (2026) note that extracellular respiration provides access to conductive minerals and other external redox partners, making such pathways ecologically plausible in ancient anoxic environments. Plausibility, however, does not demonstrate that modern EET systems derive from a single ancestral respiratory module followed by widespread loss. Recent comparative work instead shows that functionally similar trans‐outer‐membrane EET pathways can be phylogenetically unrelated. Shaw et al. (2025) identified metal‐reducing, outer‐membrane‐cytochrome, and porin‐cytochrome pathways with distinct evolutionary origins in Desulfobacterota, including co‐expression of phylogenetically distant systems under EET growth conditions. These observations support evolutionary plurality: retained ancestral functions, lineage‐specific loss, horizontal acquisition, and independent innovation may all have contributed to the present distribution of electroactivity.

**FIGURE 1:**
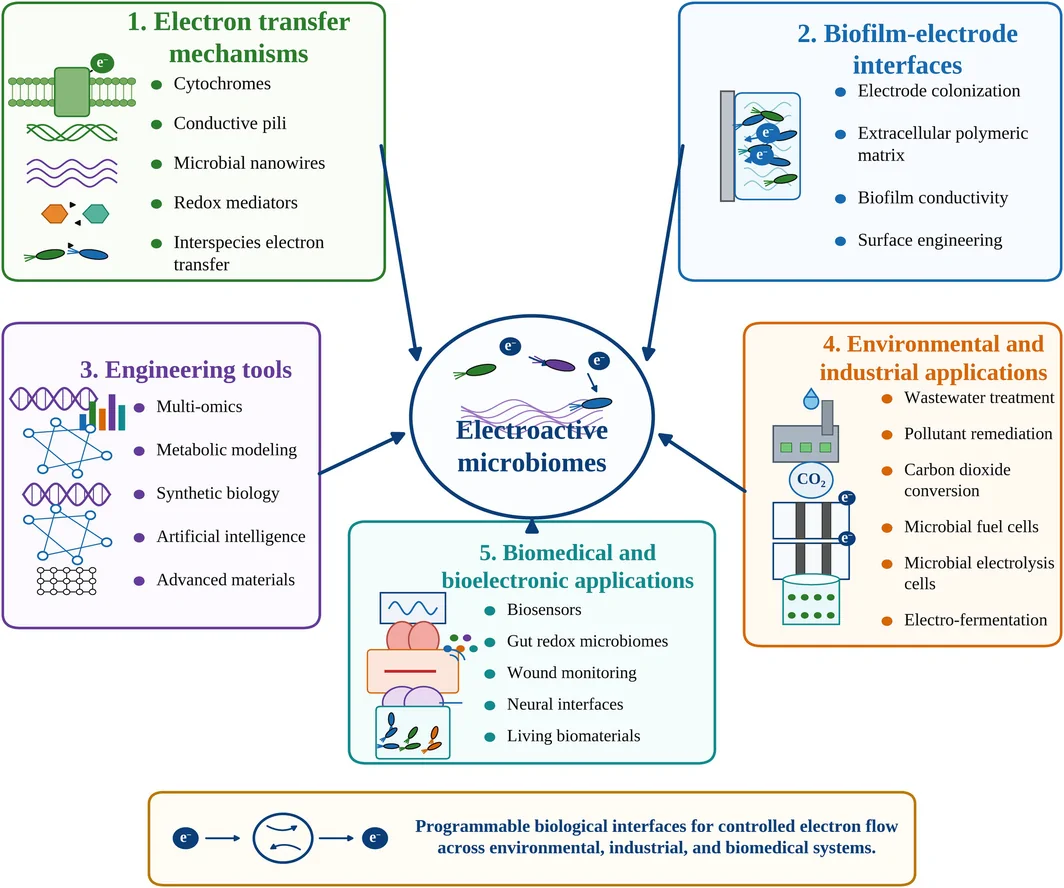
Integrative framework for electroactive microbiomes linking electron‐transfer mechanisms, biofilm–electrode interfaces, engineering tools, and application domains.

Horizontal gene transfer can contribute to EET evolution, but its role should be stated at the level supported by comparative genomics. Baker et al. (2022) identified the MtrCAB conduit across phylogenetically diverse bacteria and found patterns consistent with both horizontal and subsequent vertical transmission. This provides direct evidence for dissemination of a specific EET module, not for universal mobility of all cytochrome‐ or pilin‐associated genes. The wider distribution of electroactive capacity therefore cannot be inferred from the presence of individual redox genes alone. Comparative phylogenomics should combine conserved synteny, gene‐tree/species‐tree reconciliation, genomic context, expression under EET‐selective conditions, and functional validation. The alternative possibility that uncultivated lineages retain unexpressed or condition‐dependent electroactive traits also remains unresolved (Zhou, Zhang, et al. 2023). Consequently, predictions of electroactive potential should distinguish demonstrated EET machinery from genomic potential and should accommodate multiple evolutionary routes rather than assuming either a single ancestral origin or pervasive horizontal transfer (Figure 1).

## 4 Extracellular Electron Transfer Mechanisms

### 4.1 Direct Electron Transfer

Direct electron transfer describes extracellular electron exchange without a freely diffusible mediator between the cell and the external donor or acceptor. Its principal advantage is not an intrinsically higher rate than mediated transfer, but the possibility of sustained electron exchange without continuous mediator production or addition; actual rates remain organism‐, interface‐, and condition‐dependent. In model systems, direct transfer is associated principally with multiheme *c*‐type cytochromes and conductive appendages or filaments. Studies of *Geobacter sulfurreducens* have revealed extensive cytochrome networks whose deployment changes with electron donor and acceptor conditions (Ueki 2021). In cytochrome filaments such as OmcS, adjacent hemes can approach edge‐to‐edge separations of approximately 3.5–6 Å, providing a structural basis for electron hopping along the protein (Wang et al. 2019). Conductive pili have also been proposed to support longer‐range transport. In *G. sulfurreducens* , manipulation of aromatic residues in PilA‐associated structures has produced large changes in measured conductivity (Lovley 2017; Guberman‐Pfeffer et al. 2024), but these results must be interpreted alongside continuing debate about which extracellular filaments dominate under particular growth and preparation conditions. Direct transfer is therefore best treated as a family of protein‐ and structure‐mediated routes whose performance depends on molecular composition, spatial organisation, electrode potential, and biofilm state.

The principal structural controversy concerns the identity of conductive filaments in *G. sulfurreducens* . Cryo‐electron microscopy at 3.7 Å resolution showed that filaments purified from electrode‐grown cells could consist of polymerised hexaheme OmcS, with stacked hemes forming a plausible conduction pathway (Wang et al. 2019). This result challenged a simple model in which PilA‐based pili are the universal long‐range conduit. Conversely, atomic‐force microscopy of intact cells reported that most observed filaments were approximately 3 nm in diameter and were consistent with electrically conductive pili, whereas a smaller fraction had OmcS‐like morphology (Liu et al. 2021). A strain lacking several abundant outer‐surface cytochromes also retained 3 nm conductive filaments. These apparently conflicting results are important rather than merely contradictory because purification method, growth condition, strain background, filament abundance, and measurement scale can select different extracellular structures. Current evidence therefore supports multiple conductive components whose relative contribution can change with experimental context; it does not justify assigning all micrometre‐scale electron transport in *Geobacter* biofilms to a single filament type.

**FIGURE 2:**
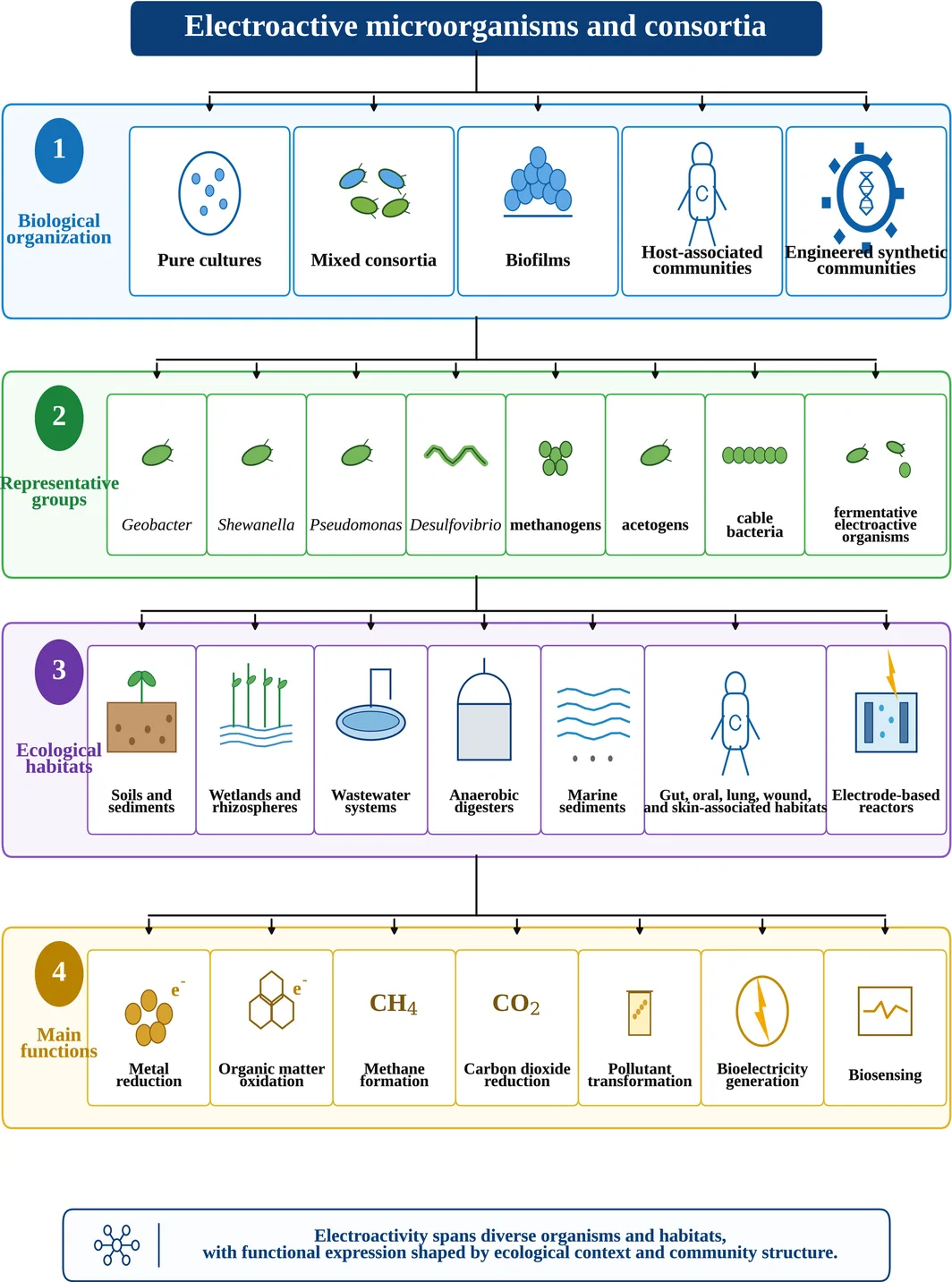
Evidence‐aware classification and ecological distribution of electroactive microorganisms and consortia.

Generalisation beyond model organisms requires an equally cautious standard. The putative pilin from *Syntrophus aciditrophicus* produced conductive filaments when heterologously expressed in *G. sulfurreducens* (Walker et al. 2020). This demonstrates a conductive phenotype in the engineered *Geobacter* host, but it does not establish that native *S. aciditrophicus* expresses conductive pili or performs direct interspecies electron transfer under physiological conditions. Native capacity would require evidence of expression and assembly in *S. aciditrophicus* together with direct electrochemical or partner‐dependent electron‐flux measurements. Other taxa illustrate genuinely different long‐range strategies: cable bacteria conduct electrons over centimetre scales through specialised intracellular fibre structures (Hiralal et al. 2024), while *Methanospirillum hungatei* produces a conductive archaellum with a structural organisation distinct from canonical bacterial cytochrome conduits (Guberman‐Pfeffer et al. 2024). These examples show that direct EET is mechanistically plural. Observations from *G. sulfurreducens* or *S. oneidensis* should therefore not be transferred automatically to undefined mixed communities, and mechanism assignment should rely on structural, genetic, electrochemical, and physiological evidence within the system being studied (Figure 2).

### 4.2 Indirect Electron Transfer

Indirect electron transfer addresses a central constraint in electroactive microbiomes: many electron acceptors, including electrodes, minerals, pollutants, humic matrices, and extracellular redox‐active compounds, are spatially separated from intracellular electron‐generating metabolism and cannot be reduced efficiently through direct cell contact alone (Roy et al. 2026). This mechanism therefore relies on electron shuttles, redox mediators, and soluble conductive metabolites that cycle between oxidised and reduced states, allowing electrons produced by microbial metabolism to be transferred across the extracellular space to terminal acceptors. Established examples include endogenous flavins, phenazines, quinones, riboflavin‐like compounds, and exogenous or environmental mediators such as humic substances, neutral red, ferricyanide, AQDS, anthraquinone derivatives, and biochar‐associated redox moieties (Zhang et al. 2023). The strength of this pathway lies in its ability to overcome the spatial and permeability limitations of direct transfer, particularly when pollutants or macromolecular electron acceptors cannot enter cells, or when electron exchange must occur across sediments, aggregates, electrodes, or biofilms. In anaerobic digestion and wastewater systems, electron shuttles have been reported to accelerate pollutant reduction and resource recovery because they facilitate contact between microbial electrons and extracellular electron acceptors, while endogenous mediators such as flavins, phenazines, and quinones are especially important because they link microbial metabolism to extracellular redox cycling without requiring permanent physical attachment to the acceptor (Zhou, Li, et al. 2023; Lv et al. 2024).

**FIGURE 3:**
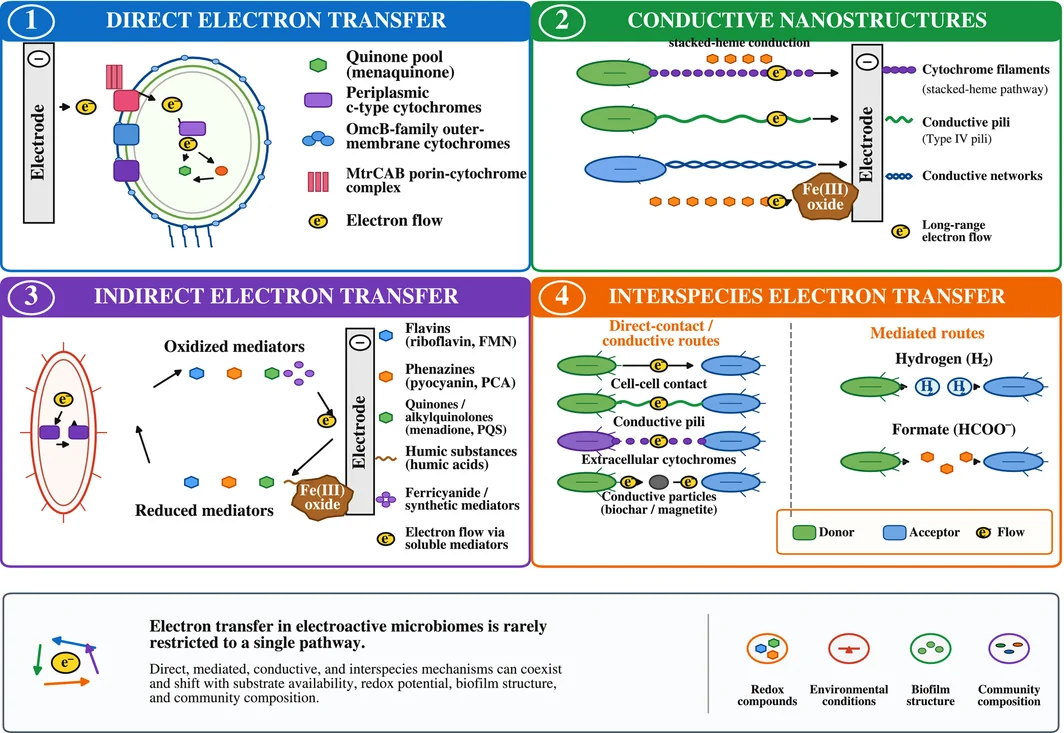
Comparative extracellular electron‐transfer routes in electroactive microbiomes. Direct cell–electrode transfer, conductive nanostructures, soluble mediator‐based transfer, and interspecies transfer differ in physical‐contact requirements, spatial range, redox chemistry, and attribution uncertainty; multiple routes can coexist within the same biofilm.

However, the mechanistic value of indirect transfer is not only its ability to increase current or degradation rates, but also its capacity to redistribute electrons across complex systems. This is evident in humic‐substance‐mediated denitrification, where humic substances act as abundant natural electron shuttles in soils, sediments, and waters, support long‐distance indirect electron transfer, and contribute to nitrate reduction and N_2_O mitigation through cytochrome‐associated pathways (Song et al. 2025). The same principle appears in microbial electrosynthesis, where neutral red and 2‐hydroxy‐1,4‐naphthoquinone improved acetate production from CO_2_ compared with mediator‐free controls, while hydroquinone reduced acetate production, and mediator addition lowered electron and carbon recovery efficiency because electrons were diverted into competing reduction pathways (Li et al. 2020). Similarly, in biophotovoltaic systems, ferricyanide supported longer‐term current output in *Synechocystis* sp. PCC 6803, whereas benzoquinone and cobalt‐based mediators generated higher short‐term currents but disrupted photosynthetic electron flow and cell growth (Yuan, Bai, et al. 2025). These contrasting outcomes show that mediator‐based transfer is not a universally positive enhancement strategy; its performance depends on redox potential, molecular size, diffusivity, stability, toxicity, concentration, interaction with cellular electron transport chains, and competition between target and non‐target electron sinks. Recent work on low‐molecular‐weight humic fractions further narrows this interpretation by showing that quinone‐rich fractions can accelerate microbial ferrihydrite reduction and that electron shuttling has a measurable spatial limit, reported as a critical distance of 117.2 nm for low‐molecular‐weight Leonardite humic acid fractions (Xue et al. 2025). Thus, indirect electron transfer is best understood as a flexible but chemically constrained electron‐distribution strategy. It expands the functional range of electroactive microbiomes, but its predictability depends on aligning mediator chemistry with microbial physiology, target redox reactions, and system architecture (Figure 3).

### 4.3 Interspecies Electron Transfer

Interspecies electron transfer extends extracellular respiration from individual cells to community‐level redox cooperation, where electrons released by fermentative, acetogenic, or exoelectrogenic organisms must be consumed efficiently by methanogens, denitrifiers, sulphate reducers, or other partners (Desmond‐Le Quéméner et al. 2021). In anaerobic digestion, classical interspecies transfer through H_2_ or formate is highly sensitive to hydrogen partial pressure, intercellular distance, and electron‐consumption kinetics (Su et al. 2023). Direct interspecies electron transfer (DIET) describes electron exchange between partners without H_2_ or formate serving as the principal diffusible carrier and can involve conductive pili, outer‐surface *c*‐type cytochromes, conductive extracellular structures, close cell association, or conductive materials (Zhuang et al. 2022). DIET can reduce dependence on diffusional carriers in compatible consortia, but it should not be assumed to be universally faster or thermodynamically superior. Relative contributions depend on partner identity, substrate, redox conditions, aggregate architecture, conductive interfaces, and the availability of competing transfer routes.

Conductive‐material additions to anaerobic digestion often increase methane production, shorten lag phases, or improve stability, and biochar, activated carbon, carbon cloth, magnetite, and related materials have repeatedly been associated with stronger syntrophic performance (Wu et al. 2020; Valentin et al. 2023; Worajo et al. 2025). These process responses are consistent with facilitated interspecies electron exchange but are not, by themselves, proof of DIET because the same materials can retain biomass, adsorb inhibitors, buffer local chemistry, alter microbial aggregation, provide trace elements, or modify hydrogen‐transfer microenvironments (Pilarska 2026). Conventional H_2_/formate‐mediated syntrophy can also respond to changes in intercellular distance, local chemistry, and carrier turnover, so a material‐induced process improvement does not uniquely identify a direct electrical route (Su et al. 2023). Recent mixed‐community studies further indicate that multiple interspecies transfer strategies can coexist or shift with substrate and reactor conditions (Liu et al. 2025; Zhao et al. 2025). Strong mechanistic attribution therefore requires convergent evidence: compatible partner association, expression or necessity of relevant EET machinery, aggregate or material conductivity, electrochemical behaviour, perturbation experiments, and process responses that cannot be adequately explained by H_2_/formate transfer or non‐electrical material effects. The distinction is important because reactor enhancement and mechanism identification are related but separate claims.

Community electron sharing also extends beyond a binary DIET‐versus‐H_2_/formate model. Humic substances can mediate electron exchange between EET‐capable organisms and denitrifiers in sediments (Song et al. 2025), while extracellular matrix components can organise the close spatial architecture needed for efficient syntrophy even when the matrix itself is not the principal conductor (Zhuang et al. 2022). In mixed communities, diffusible mediators, H_2_/formate transfer, direct cell‐to‐cell transfer, and conductive‐material‐assisted exchange can coexist and their relative contributions can shift with substrate, community composition, redox gradient, aggregate structure, and material properties. For this reason, improved methane production or co‐enrichment of putative DIET taxa should be treated as reactor‐level evidence unless direct electron‐flux or pathway‐specific experiments establish the mechanism. The practical transition is from labelling communities by a presumed transfer route to quantifying which routes carry electron flux under defined operating conditions (Table 1).

**TABLE 1:** Core extracellular electron transfer mechanisms and representative mechanistic evidence in electroactive microbiomes.

Electron‐transfer mode	Experimental basis	Principal components	Mechanistic inference	Comparative value	Key limitation	References
Cytochrome‐mediated direct electron transfer	*S. oneidensis* MR‐1 Fe(III)‐respiration mutagenesis and *G. sulfurreducens* structure–function studies	MtrA/MtrB/MtrC and other multiheme *c*‐type cytochromes	Heme spacing, folding, and porin–cytochrome continuity control trans‐envelope electron flux.	Direct molecular route to insoluble acceptors and electrodes; genetically tractable.	Strongly dependent on correct protein assembly and partner interactions.	(Campbell et al. 2022; Zakizadeh Tabari and Hochbaum 2025)
Cytochrome‐filament nanowires	Cryo‐EM of *Geobacter* OmcE/OmcS/OmcZ filaments	Polymerised cytochromes; stacked hemes	Polymerised cytochromes can support long‐range conduction.	Extends EET beyond the cell surface.	Filament identity varies with strain and growth condition; generalisation remains limited.	(Wang et al. 2023, 2022)
Engineered conductive protein nanowires	Genetic functionalisation of *Geobacter* conductive nanowire monomers	Conductive protein nanowires; peptide tags	Surface functionality can be added while retaining conductivity.	Programmable sensing and material interfaces.	Assembly, conductivity, and device durability must remain stable.	(Ueki et al. 2019)
Biofilm‐mediated electron transport	*Geobacter* biofilms on electron‐accepting surfaces	Pili, *c*‐type cytochromes, extracellular matrix	Spatially organised matrix supports electron transport across multilayer biofilms.	Cells beyond direct electrode contact can contribute.	Conductivity and activity are spatially heterogeneous; diffusion limits can dominate.	(Reguera 2018)
Mediated electron transfer	MFC consortia with endogenous or added redox shuttles	Flavins, phenazines, quinones, and other mediators	Diffusible shuttles transfer electrons without continuous physical contact.	Useful in porous, heterogeneous, or weakly colonised systems.	Mediator loss, toxicity, side reactions, and diffusion constraints.	(Mishra et al. 2026)
Direct interspecies electron transfer	Defined *Geobacter*–*Geobacter* and *Geobacter*–*Methanosarcina* cocultures	Outer‐surface *c*‐type cytochromes, conductive pili, syntrophic partners	Electron‐transfer route is partner‐specific and can alter donor physiology.	Can reduce dependence on H_2_/formate diffusion in compatible consortia.	Relative rates are partner‐ and condition‐specific; attribution is difficult in mixed communities.	(Holmes et al. 2022)
Conductive‐material‐assisted interspecies transfer	Anaerobic digesters amended with biochar, activated carbon, or magnetite	Conductive particles, fermenters, and methanogens	Materials can bridge partners and promote aggregation.	Can improve methane production and stress resilience.	Performance gains do not by themselves prove DIET; adsorption, buffering, and biomass retention can contribute.	(Valentin et al. 2023; Chen et al. 2022)
Biogenic‐mineral‐assisted interspecies transfer	Low‐strength sewage reactor with magnetotactic bacteria and biogenic magnetite	Magnetotactic bacteria, magnetite, *Geobacter*, *Methanobacterium*	Biogenic minerals may contribute to electron‐sharing networks.	Potential endogenous conduit without added conductive material.	Emerging mechanism; co‐occurrence and gene expression do not establish electron flux.	(Dornelles et al. 2026)
Electrode‐assisted extracellular electron uptake	MES biocathodes reducing CO_2_ to acetate‐dominated products	Cathodic biofilms, acetogens, electrotrophs, Wood–Ljungdahl pathway	Cathode electrons enter cells directly or through intermediates and support carbon fixation.	Links renewable electricity to chemical production.	Uptake pathways, rate, selectivity, energy balance, and separation remain limiting.	(LaBelle et al. 2020; Bañeras et al. 2024)
Cathodic electromethanogenesis	Methanogenic biocathodes under controlled cathode potentials	Methanogens, cathode interface, H_2_, and inward‐electron pathways	CO_2_‐to‐CH_4_ can proceed by direct uptake and/or cathodically generated H_2_.	Relevant to electricity storage and biogas upgrading.	Mechanism and rate depend strongly on potential, material, contact, and competing H_2_ pathways.	(Mayer et al. 2022; Cai et al. 2024)

## 5 Electroactive Biofilms and Microbiome‐Electrode Interfaces

Electroactive biofilms form the living reaction layer of microbial electrochemical systems by connecting microbial metabolism with solid electrodes (Brožková et al. 2022). Their function extends beyond attachment: biofilms determine which cells remain active, how substrates and protons diffuse, how redox proteins and mediators are retained, and how electron‐transfer pathways are spatially organised. Biofilm formation progresses from reversible contact to irreversible adhesion, microcolony development, maturation, and dispersal under the combined influence of hydrodynamics, surface chemistry, microbial physiology, quorum sensing, and cyclic‐di‐GMP signaling (Conners et al. 2022; Paquete 2026). Extracellular polymeric substances (EPS) form the matrix that anchors cells, creates diffusion pathways, retains redox‐active components, and supports community organisation. EPS can enhance electroactivity when it stabilises conductive or redox‐active structures, but excessive or poorly conductive matrix material can increase transport resistance or separate cell‐surface cytochromes from the electrode (Roy et al. 2022). Thus, EPS abundance is not inherently beneficial; composition, redox activity, thickness, and spatial distribution determine whether it supports or constrains EET.

Biofilm thickness illustrates why electrochemical performance cannot be inferred from biomass alone. Electroactive biofilms develop internal gradients in substrate, proton concentration, redox potential, metabolites, and electron‐transfer opportunity, and these gradients can create spatially distinct active and inactive regions (Conners et al. 2022; Paquete 2026). In a *Geobacter sulfurreducens* system summarised in the cited evidence, electrochemical activity was highest at a biofilm thickness of approximately 20 μm and declined as the biofilm approached about 45 μm because inactive regions and transport resistance increased (Paquete 2026). These values are specific to that organism, electrode, medium, and operating configuration; they should not be treated as a universal optimum for mixed‐community reactors. Proton accumulation provides an additional constraint because electrons can move towards the anode while protons generated within the biofilm must diffuse outward; poor buffer penetration and local acidification can therefore suppress current even when the biofilm remains physically attached (Paquete 2026). The relevant design objective is a functionally organised biofilm that maintains active biomass, electron conduction, substrate access, and proton removal, not maximum thickness.

Electron transport within biofilms is mechanistically diverse. Direct electron transfer occurs when cells exchange electrons with the electrode through outer‐membrane multiheme *c*‐type cytochromes, conductive pili, microbial nanowires, or redox‐active extracellular matrices. Indirect electron transfer occurs when soluble shuttles, humic substances, flavins, phenazines, or other mediators carry electrons between cells and the electrode. Some microorganisms rely mainly on one route, while others combine several pathways depending on growth conditions and electron acceptor availability (Conners et al. 2022; Roy et al. 2022). *Shewanella oneidensis* and *Geobacter sulfurreducens* remain central model organisms because studies on these bacteria have clarified the roles of Mtr/Omc cytochromes, conductive pili, and nanowires in electrode‐linked respiration (Conners et al. 2022). Yet practical systems rarely operate as pure cultures. Mixed‐species biofilms can process more complex substrates, tolerate changes in pH and temperature, and create cooperative microenvironments through cross‐feeding, redox mediator exchange, oxygen scavenging, and shared biofilm protection (Paquete 2026). This benefit also has limits. Non‐electroactive organisms may compete for substrates, divert electrons towards methane or other products, release inhibitory metabolites, or reduce electrode‐focused electron recovery. Microbiome‐electrode interfaces should therefore be viewed as ecological‐electrochemical systems. Current output reflects not only the electroactivity of a dominant species but also consortium structure, metabolic division of labour, and the balance between cooperative and competitive interactions.

Electrode materials and surface engineering determine whether this living interface becomes productive or poorly connected. An effective electrode must combine high conductivity, low resistance, low impedance, chemical stability, large accessible surface area, and biocompatibility. It must also support microbial adhesion, growth, and electron exchange without causing toxicity or excessive background reactions. Carbon‐based electrodes, including graphite, carbon felt, carbon cloth, carbon paper, charcoal foam, and carbon brushes, are widely used because they provide favourable conductivity, chemical stability, microbial compatibility, and surface features that support colonisation and extracellular electron transfer (Roy et al. 2022; Lahiri et al. 2022). Other materials, including titanium, stainless steel, nickel foam, ceramics, and fluorine‐doped tin oxide systems, have also been explored, but their performance depends strongly on surface chemistry and morphology. Titanium is corrosion resistant, but its low bioactivity and conductivity can limit biofilm performance unless its surface is functionalised, for example, with TiO_2_ nanotubes. Ceramic and FTO‐coated porous materials can support selected photosynthetic biofilms, while some graphite‐based fibres may not support strong attachment for particular organisms (Conners et al. 2022; Roy et al. 2022). Surface engineering is therefore not a minor material adjustment. It changes how cells attach, how extracellular structures align, how charge crosses the interface, and how much of the electrode becomes biologically active.

**FIGURE 4:**
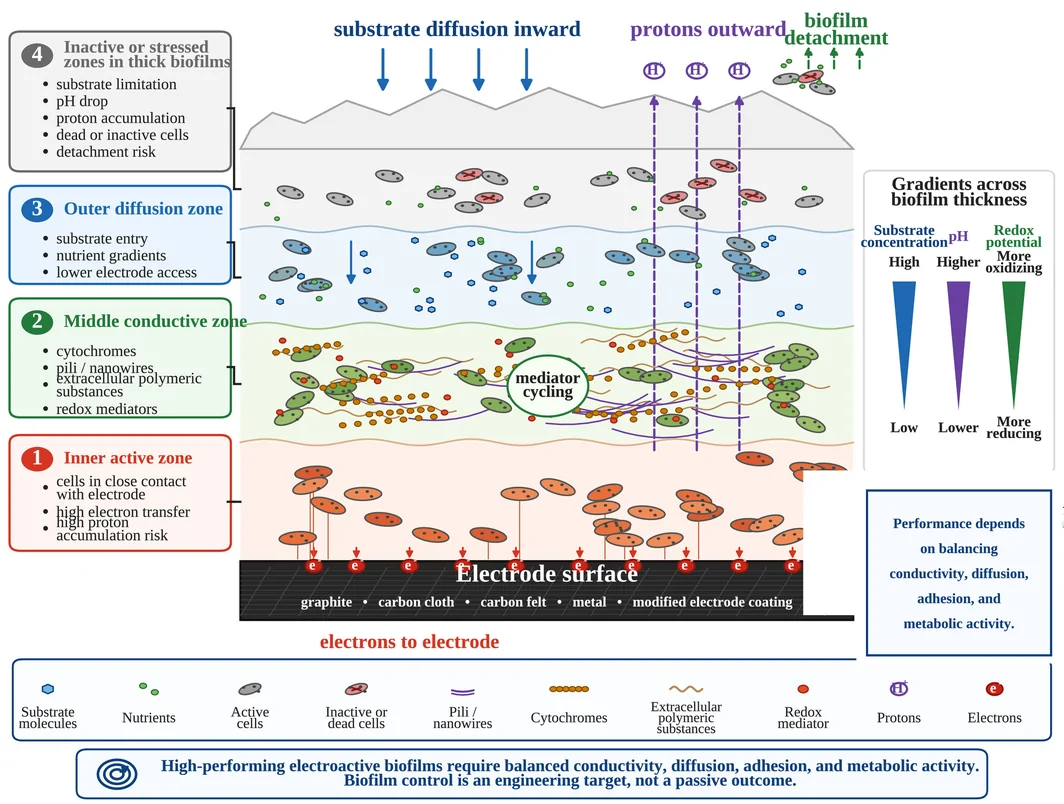
Electroactive biofilm–electrode interface showing substrate and proton gradients, active and inactive zones, mediator cycling, conductive structures, and biofilm detachment.

Biointerface optimisation requires biological, material, and operational control at the same time. Quorum‐sensing or quorum‐quenching approaches can alter EPS production, attachment, and biofilm thickness, showing that biological regulation changes the electrochemical interface rather than simply changing biomass quantity (Paquete 2026). Genetic or strain‐level changes can likewise redistribute active cells and electron‐transfer components, but a greater abundance of an EET component does not automatically produce greater whole‐biofilm current when diffusion or architecture is limiting (Conners et al. 2022; Paquete 2026). Material modification and microbiome‐oriented interface engineering provide an additional control layer by changing adhesion, local conductivity, redox accessibility, and electron uptake (Paquete 2026; Roy et al. 2022). Quantitative improvements reported in these studies are most informative as within‐study effect sizes, not transferable performance benchmarks, because organism or inoculum, electrode projected area and morphology, substrate loading, reactor geometry, polarisation protocol, operation time, and normalisation basis differ among experiments. The consistent inference is mechanistic rather than numerical: performance improves when microbial activity, biofilm architecture, electrode surface chemistry, substrate diffusion, proton transport, and electron conduction are aligned. The microbiome–electrode interface should be treated as an ecological‐electrochemical system whose optimum is configuration‐specific (Figure 4).

## 6 Multi‐Omics and Computational Engineering of Electroactive Microbiomes

Multi‐omics and computational engineering are most useful when they reduce uncertainty about which members and pathways actually carry electron flux in mixed electroactive communities. Genomics and metagenomics identify functional potential; transcriptomics and proteomics identify condition‐dependent expression; electrochemical measurements and physiology are required to connect that potential and expression to EET function. Metabolic models can expose carbon and electron‐allocation constraints, while artificial intelligence and machine learning can identify multivariate performance patterns or prioritise experiments. None of these layers alone proves a mechanism. This section therefore emphasises electroactive‐system‐specific studies and treats computational outputs as experimentally testable hypotheses rather than substitutes for functional validation.

### 6.1 Genomics and Metagenomics

**FIGURE 5:**
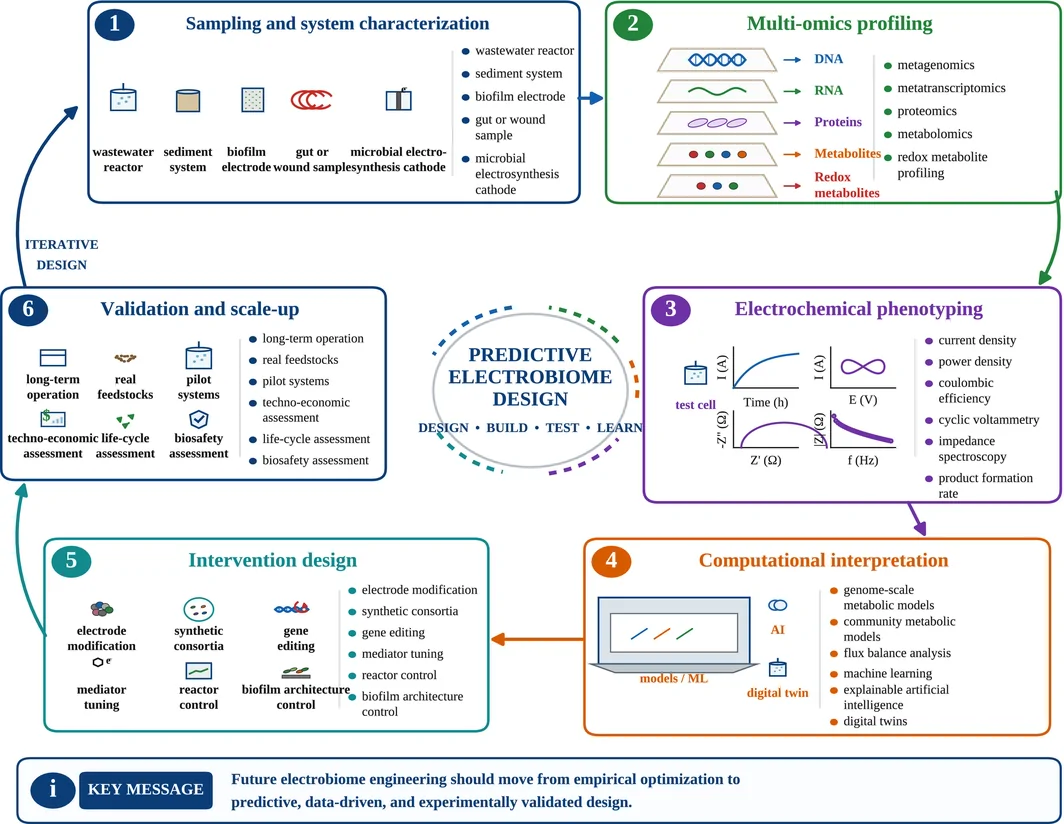
Predictive electroactive‐microbiome engineering workflow linking system characterisation, multi‐omics profiling, electrochemical phenotyping, computational interpretation, intervention design, and experimental validation.

Genomics and metagenomics define the genetic inventory available for EET, substrate conversion, biofilm formation, stress tolerance, and interspecies interaction. Their value is strongest when sampling and electrochemical conditions are linked directly. Godain et al. (2023) combined metagenomics and metaproteomics in microbial fuel cells and found that an applied start‐up voltage shifted both community composition and expressed electron‐transfer signatures, with stronger direct cytochrome‐associated features under the applied condition and broader mediator‐associated participation without it. Genome‐centric metagenomics can resolve community‐level electron‐sharing networks at still finer resolution. Jovicic et al. (2026) reconstructed a conductive‐particle‐dependent sediment consortium containing candidate electrogenic acetate oxidisers, a *Methanosarcina* electron‐accepting partner, and extensive predicted EET machinery. These studies illustrate both the strength and limitation of metagenomics: it can identify plausible pathway architecture and partner relationships, but gene presence does not establish electron flux. Functional claims require expression data, electrochemical behaviour, perturbation, or physiological evidence under the same operating conditions (Figure 5).

### 6.2 Transcriptomics and Proteomics

Transcriptomics and proteomics narrow the gap between genetic potential and active function. In *Geobacter sulfurreducens* biofilms, Yang, Xia, et al. (2024) combined electrochemistry, spectroscopy, and proteomics to show that anodic and cathodic EPS contained different redox‐protein signatures, supporting condition‐dependent outward and inward EET rather than a fixed pathway. Mickol et al. (2021) used metagenomics and metatranscriptomics to identify potential‐dependent expression changes in a mixed biofilm capable of both energy‐generating and energy‐storing electrode reactions, while also recognising that transcript patterns did not by themselves resolve every electron‐uptake route. At the reactor‐interface scale, Dong et al. (2024) linked electrode orientation with biofilm structure, proteomic responses, *c*‐type cytochrome abundance, community composition, and current density. Together, these studies show why expression data should be paired with current, cyclic voltammetry, impedance, metabolite measurements, microscopy, or targeted perturbation. Increased transcription or protein abundance is mechanistically informative only when it is connected to functional electron transfer in the same system.

### 6.3 Metabolic Network Modelling

Metabolic network modelling provides a quantitative framework for asking where carbon and reducing equivalents are allocated and which reactions may constrain electrode‐linked metabolism. A recent co‐culture study integrated a two‐cell genome‐scale model of *Shewanella oneidensis* MR‐1 and *Geobacter sulfurreducens* with experimentally tested medium regulation, using predicted metabolic bottlenecks to improve electron generation in a microbial fuel cell (Ghasemi Naraghi et al. 2026). Genome‐scale reconstruction of *S. oneidensis* MR‐1 has likewise been used to analyse metabolic potential under bioelectrochemical constraints (Luo et al. 2022). At the process scale, a mechanistic model of biofilm‐driven microbial electrosynthesis has linked transport, microbial growth, and carboxylate production from CO_2_ (Cabau‐Peinado et al. 2021). These examples are stronger than generic metabolic‐modelling analogies because they are anchored in electroactive organisms or reactors. Their limitation remains model dependence: annotations, exchange boundaries, biomass equations, redox assumptions, transport representations, and community interactions can materially change predictions. Modelling is therefore most defensible as an iterative cycle in which simulations identify bottlenecks, experiments test them, and measured electrochemical and omics data refine the model.

### 6.4 Artificial Intelligence and Machine Learning in Electroactive Microbiome Design

Artificial intelligence (AI) and machine learning (ML) can support electroactive‐microbiome design when relationships among microbial composition, materials, operating conditions, and performance become too multivariate for simple empirical correlations. A bioelectrochemical‐system‐specific review by Li et al. (2023) documents applications of AI and ML to reactor performance, microbial‐community information, products, and substrates while emphasising data quality and model limitations. Primary work has begun to move from retrospective fitting towards operational prediction. Hess‐Dunlop et al. (2025), for example, used deep‐learning time‐series models to forecast soil microbial‐fuel‐cell energy generation across short time horizons and quantified prediction uncertainty. Such models can help with forecasting, feature ranking, anomaly detection, or experiment prioritisation, but they do not establish causation. AI‐guided design is therefore best treated as decision support constrained by mechanistic knowledge, external validation, uncertainty analysis, and standardised input variables rather than as an autonomous replacement for electrochemistry or microbial physiology.

### 6.5 Synthetic Biology and Genome Engineering

Synthetic biology and genome engineering provide the intervention layer for testing whether identified EET bottlenecks can be altered deliberately. Zhang et al. (2024) synthesise electroactive‐microorganism‐specific strategies that target substrate utilisation, reducing‐equivalent availability, *c*‐type cytochromes, conductive structures, redox mediators, global regulation, biofilm formation, and inward electron transfer. Direct experimental evidence is available in defined strains: Fan et al. (2021) developed a high‐efficiency genome‐editing system in *Shewanella oneidensis* MR‐1 and used it to program EET‐related functions. Bird et al. (2021) place such work within the broader synthetic‐biology toolkit for native electroactive bacteria and engineered EET pathways. The evidence is more mature for defined organisms than for undefined mixed microbiomes, where ecological stability, competition, horizontal gene transfer, containment, and long‐term maintenance complicate genotype‐to‐process prediction. For environmental deployment, engineered functions therefore require not only increased current or product formation but also stability, ecological compatibility, and biosafety under realistic operating conditions.

## 7 Environmental and Ecological Applications

Electroactive microbiomes are environmentally important because they connect microbial metabolism with redox transformations that would otherwise require chemical or energy‐intensive control. Their evidence base, however, is not uniform across applications. Wastewater treatment and anaerobic digestion have the broadest reactor‐level literature and repeated demonstrations of coupled treatment, electron recovery, or methane‐process enhancement. Soil and sediment studies provide strong ecological and mechanistic evidence for electrode‐linked redox processes but fewer long‐duration field datasets. Pollutant‐remediation outcomes are contaminant‐ and configuration‐specific, while cathodic carbon conversion has growing experimental support but remains constrained by electron‐uptake attribution, production rate, selectivity, and energy balance. This maturity gradient is used below to compare applications without treating laboratory proof‐of‐principle as equivalent to field readiness.

### 7.1 Wastewater Treatment and Resource Recovery

**FIGURE 6:**
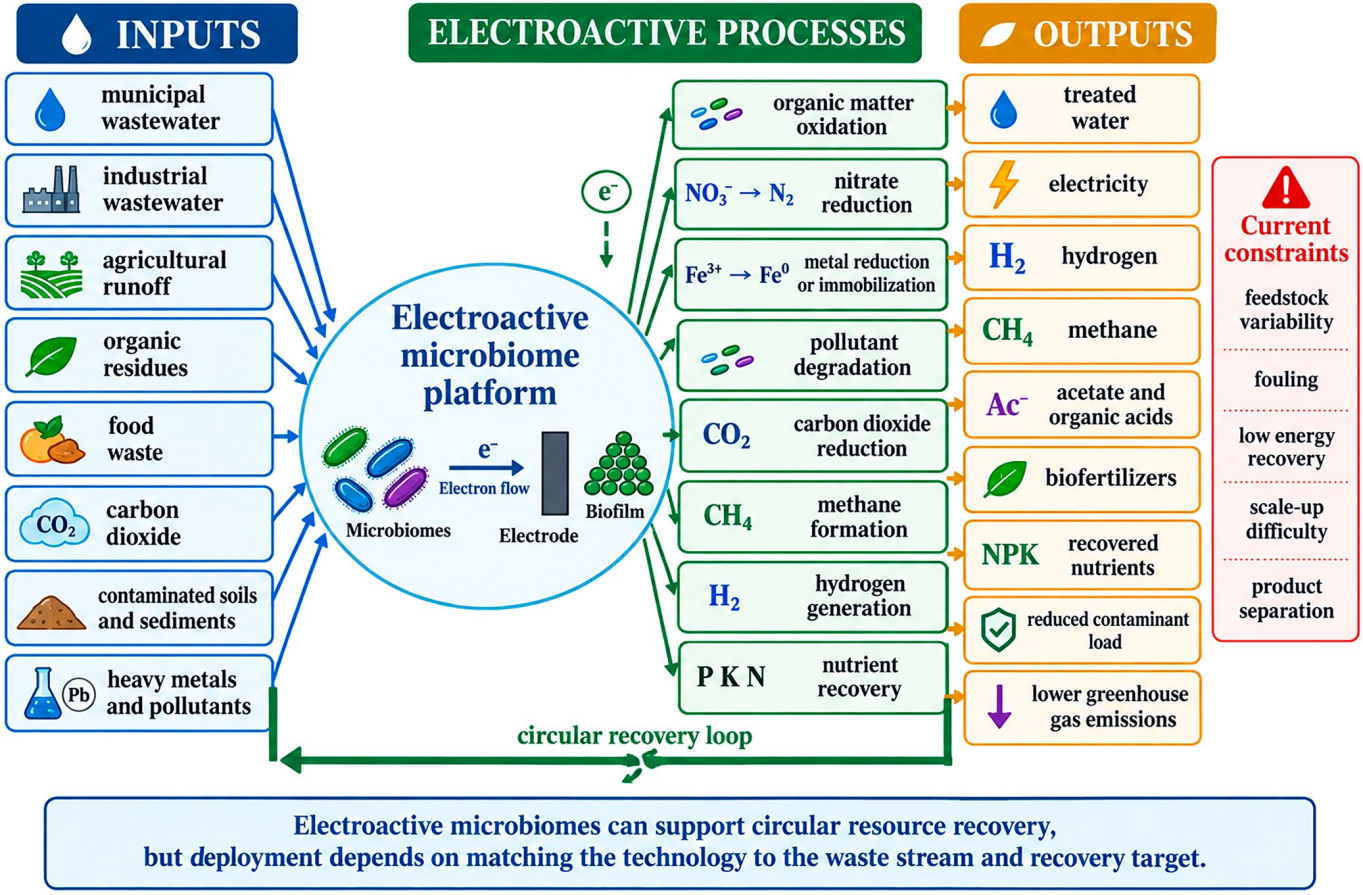
Environmental and circular‐bioeconomy functions of electroactive microbiomes.

Wastewater treatment is the most developed environmental application of electroactive microbiomes because it links organic‐matter removal with energy or resource recovery. In bioelectrochemical systems (BESs), including microbial fuel cells and microbial electrolysis cells, anode‐associated communities oxidise organic substrates and transfer electrons through direct or mediated EET. Chemical oxygen demand (COD) removal, current production, methane or hydrogen recovery, and other outputs are strongly conditioned by reactor architecture, substrate, electrode properties, loading, and community structure. This configuration dependence is evident in primary work comparing single‐ and dual‐chamber systems under simultaneous microplastic and antibiotic exposure, where reactor architecture altered wastewater‐treatment behaviour and methane‐recovery performance (Suleiman et al. 2026; Wang et al. 2025). Pilot and scale‐up studies show a different constraint set: internal resistance, cathode degradation, fouling, mass transfer, membrane cost, feed variability, and maintenance can dominate once optimised laboratory conditions are relaxed (Makhathini 2026). Consequently, high COD removal or power density from a particular bench reactor should not be generalised without its hydraulic, electrochemical, and normalisation context. For municipal wastewater, BES modules may be most realistic for polishing, sensing, nutrient recovery, low‐strength decentralised treatment, or integration with anaerobic digestion; for high‐strength industrial or agricultural streams, resource recovery can provide a stronger economic driver. The central translation criterion is durable treatment and recovery under real wastewater variability rather than peak current alone (Figure 6).

### 7.2 Bioremediation of Pollutants and Heavy Metals

Bioremediation uses the same electron‐transfer capacity for contaminants that require oxidation, reduction, immobilisation, or coupled transformation. In groundwater, bioelectrochemical platforms can establish spatially separated anodic and cathodic redox zones and have been investigated for metals, oxyanions, and organic contaminants (Cecconet et al. 2018). Emerging‐pollutant studies further show that an electrode can act as a controllable electron sink or donor while process models help interpret rate limitations (Ahmadi et al. 2023). For persistent polycyclic aromatic hydrocarbons, bioelectrochemical operation can combine microbial degradation, electrode‐driven redox control, mediator activity, and enrichment of degrading communities (Feng et al. 2025). Evidence strength nevertheless varies by contaminant and matrix. Removal efficiency alone does not demonstrate detoxification, and transformation products, metal speciation, pH, competing ions, electrode distance, and mass transport can determine whether a process is environmentally beneficial. The strongest interpretation is therefore contaminant‐specific redox management rather than a universal remediation effect. Future studies should couple electrochemical data with toxicity, transformation‐product analysis, mass balance, and long‐term contaminant fate.

### 7.3 Carbon Capture and Greenhouse Gas Mitigation

Carbon‐focused applications route cathode‐derived reducing power into methane, acetate, alcohols, carboxylates, or other products. Bioelectromethanogenesis provides direct evidence that electroactive cathodic communities can convert CO_2_ to methane (Zhang et al. 2019), while microbial electrosynthesis (MES) extends cathodic electron supply to multi‐carbon products (Mirea et al. 2025). The limiting question is not simply whether CO_2_ disappears from the gas or liquid phase but how electrons enter microbial metabolism and how much carbon and energy reach the intended product. Li and Dong (Li and Dong 2025) identify extracellular electron uptake as a central constraint and emphasise the need to combine spectroscopy, microscopy, electrochemistry, and meta‐omics to discriminate direct uptake from hydrogen‐mediated routes. Product rate, carbon recovery, coulombic efficiency, applied electrical energy, gas leakage, and downstream separation must therefore be evaluated together. Soil BES studies that suppress uncontrolled methane emissions address a different environmental objective from deliberately producing methane as an energy carrier (Yu et al. 2025). Carbon‐focused electroactive systems are best interpreted as carbon‐routing technologies whose climate value depends on system boundaries, electricity source, energy balance, product fate, and life‐cycle emissions.

### 7.4 Soil and Sediment Bioelectrochemistry

Soils and sediments are natural redox mosaics in which EET intersects with oxygen, nitrate, sulphate, iron, manganese, organic carbon, sulphide, methane, and humic substances. Electrodes or conductive phases can connect otherwise separated redox zones, but the resulting treatment footprint depends on local conductivity, contaminant transport, electrode spacing, and native community structure. Soil bioelectrochemical studies link microbial electron transfer with carbon, nitrogen, iron, and sulphur cycling (Yu et al. 2025). Primary evidence from saline petroleum‐contaminated soil demonstrates this spatial dependence directly: a U‐tube microbial fuel cell stimulated petroleum‐hydrocarbon degradation in the electrode‐influenced treatment zone, showing that electrode placement and soil transport properties can delimit remediation performance (Wang et al. 2012). These systems therefore differ from well‐mixed wastewater reactors. Their ecological relevance is high, but field translation requires longer operation, spatially resolved chemistry and microbiology, contaminant mass balance, and monitoring of unintended shifts in metal mobility or greenhouse‐gas production.

### 7.5 Sustainable Circular Bioeconomy Systems

Circular bioeconomy applications bring the previous sections together. The core idea is to replace linear waste management with systems that recover carbon, electrons, nutrients, and materials from waste streams. Electroactive microbiomes fit this model because they can convert wastewater, organic residues, food waste, industrial effluents, CO_2_, and biomass‐derived substrates into electricity, hydrogen, methane, carboxylates, biofuels, fertilisers, and chemical precursors. Studies on BESs for circular bioeconomy describe these platforms as waste‐to‐energy and waste‐to‐chemical systems because they can remove wastewater pollutants while generating recoverable products (Jung et al. 2020). Studies on energy and nutrient recovery from municipal and industrial waste also argue that carbon‐rich waste streams, including municipal solid waste, industrial residues, agricultural by‐products, seaweed residues, food waste, brewery wastewater, winery wastewater, confectionery waste, and dairy wastewater, should be treated as feedstocks for biorefining rather than disposal burdens (Rachbauer et al. 2024). This broader bioeconomy framing is useful because electroactive systems alone will not valorise all waste fractions. Their strongest role may be as modules within integrated biorefineries, alongside anaerobic digestion, fermentation, composting, gas fermentation, nutrient recovery, and biochar or fertiliser production. Organic waste bioconversion studies point in the same direction. Efficient circular systems require attention to feedstock variability, pretreatment, pH, temperature, carbon‐to‐nitrogen ratio, organic loading rate, hydraulic retention time, downstream processing, microbial community dynamics, techno‐economic analysis, and life‐cycle assessment (Reddy et al. 2026). Food waste valorisation further illustrates why circularity cannot be reduced to energy generation alone. Food waste can be converted into bioactive compounds, bioenergy, engineered biochars, food additives, and other renewable resources, supporting zero‐waste and resource‐efficiency goals (Pal et al. 2024). For electroactive microbiomes, the main opportunity is therefore strategic integration. They can recover electrons from dilute wastewaters, polish effluents, support decentralised power generation, assist nutrient recovery, drive CO_2_ upgrading, and improve redox control in hybrid treatment chains. Their limitation is economic concentration. Many waste streams are dilute, variable, or geographically dispersed, and the value of recovered electricity alone is often too low to justify full‐scale deployment. The circular bioeconomy case becomes stronger when multiple benefits are counted together: treatment, avoided aeration, reduced sludge, nutrient recovery, product formation, greenhouse gas mitigation, monitoring, and local resource reuse. The field should therefore move beyond asking whether electroactive microbiomes can generate power from waste. The stronger question is where their redox capabilities add unique value inside integrated, resilient, and economically realistic circular systems.

## 8 Energy and Industrial Biotechnology Applications

Energy and industrial applications share the same EET mechanisms but differ substantially in their engineering objective and maturity. Microbial fuel cells (MFCs) have the longest history of coupling organic‐matter oxidation to electrical current, yet low recoverable power and cathodic losses limit their role as stand‐alone energy devices. Microbial electrolysis cells (MECs) add external voltage to drive hydrogen, methane, or other reduced products, making energy balance and product value decisive. Electro‐fermentation (EF) uses electrodes mainly to steer redox balance and product distribution, whereas microbial electrosynthesis (MES) supplies cathodic electrons for carbon fixation and chemical production. Cross‐platform numerical comparison is therefore meaningful only when current, power, product rate, substrate conversion, applied voltage, electrode area, reactor volume, and energy recovery are reported on compatible bases. The following sections compare each platform according to its specific function rather than treating all bioelectrochemical outputs as a common performance metric.

### 8.1 Microbial Fuel Cells

**FIGURE 7:**
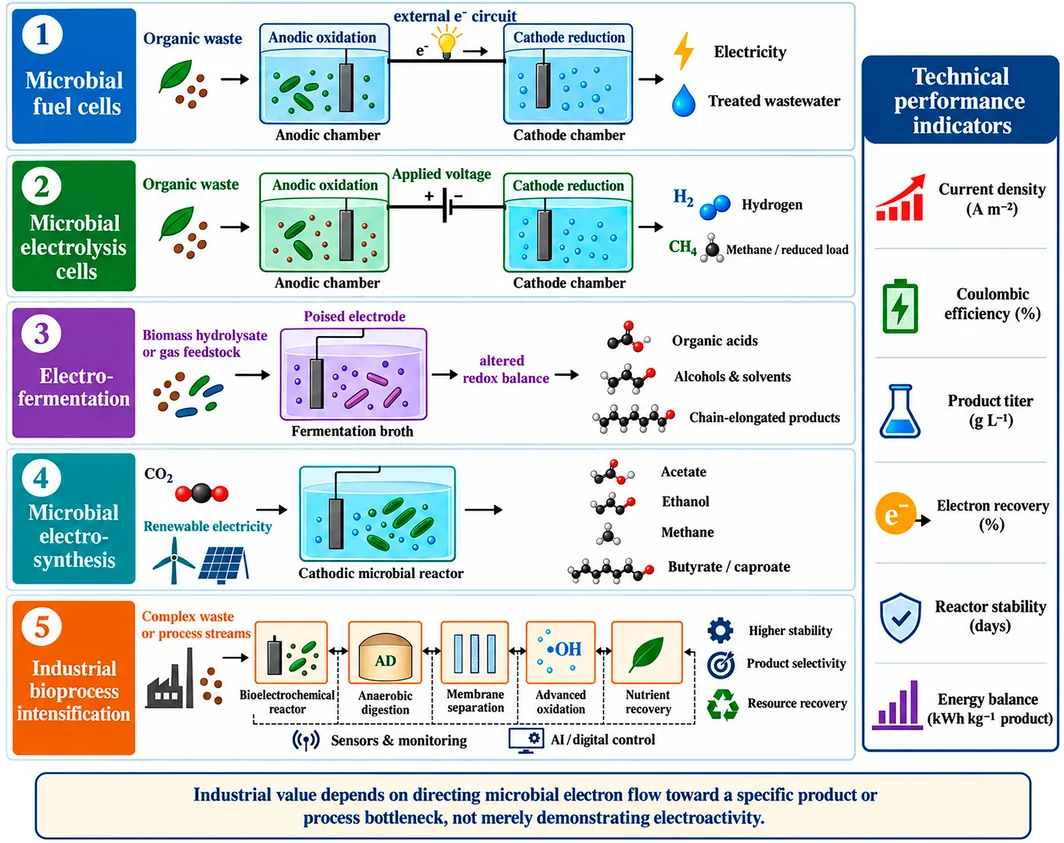
Comparative energy and industrial biotechnology platforms using electroactive microbiomes.

Microbial fuel cells are the most established electricity‐generating application of electroactive microbiomes, but whole‐cell performance reflects microbial EET, ionic transport, electrode kinetics, and reactor resistance simultaneously. At the anode, electroactive communities oxidise organic substrates and transfer electrons to the external circuit; at the cathode, oxygen reduction or another terminal reaction must sustain the corresponding ionic and charge balance. Cathode transport can itself become a limiting process. Wang et al. (2014) demonstrated that quaternary‐ammonium modification of activated‐carbon air cathodes could accelerate hydroxide transport, illustrating that a change outside the microbial biofilm can materially alter overall MFC performance. Interfacial and electrode‐design principles remain important across microbial electrochemical platforms, including systems intended for cathodic synthesis, because microbial catalysis cannot compensate indefinitely for ionic or electrochemical transport losses (Wang et al. 2014; Sonawane et al. 2021). Wastewater‐focused MFC studies with electroactive bacteria provide direct examples of simultaneous pollutant degradation and electrical recovery under defined electrode configurations (Mkilima et al. 2025b), while hybrid MFC–advanced‐oxidation systems illustrate how current‐generating biology can be integrated with a complementary treatment step for more recalcitrant wastewaters (Mkilima et al. 2025a). Reported power densities should therefore be interpreted together with cathode chemistry, projected electrode area, internal resistance, substrate loading, reactor configuration, and polarisation method. MFCs remain valuable for simultaneous treatment, sensing, and low‐power energy recovery, but their environmental value is more defensible when electricity is treated as one co‐benefit within an integrated treatment objective rather than as a universal substitute for conventional power generation (Figure 7).

### 8.2 Microbial Electrolysis Cells

Microbial electrolysis cells extend anode‐based microbial oxidation by applying external voltage to drive thermodynamically unfavourable cathodic reactions such as hydrogen evolution or electromethanogenesis. Applied voltage therefore acts both as an energy input and as an ecological‐electrochemical control variable. Ndayisenga et al. (2023) showed that changing applied voltage in a single‐chamber, membrane‐free system treating recalcitrant straw‐derived waste altered biofilm viability and hydrogen production. The reported values are specific to that reactor architecture, feed, electrode configuration, and 0.8 V operating condition and are not directly comparable with volumetric hydrogen rates, COD‐normalised yields, or energy efficiencies from other MEC designs. Studies of electromethanogenesis likewise show that cathode material, hydrogen evolution, direct or indirect electron transfer, biofilm formation, and methanogenic pathway stability can all influence methane production (Pawar et al. 2022). Comparisons of MFCs and MECs on the same wastewater further illustrate that the platforms solve different problems: MFCs prioritise electrical current, whereas MECs trade electrical input for reduced products such as hydrogen (Bin Abu Sofian et al. 2025). MEC viability must therefore be judged by net energy and product recovery, not product formation alone.

### 8.3 Electro‐Fermentation Technologies

Electro‐fermentation addresses a long‐standing limitation of conventional fermentation: microbial product formation is constrained by intracellular redox balance. In many fermentations, the product spectrum is shaped by the need to regenerate NAD^+^ or NADH, dispose of excess electrons, maintain pH, and balance carbon flux. Conventional processes manage this through aeration, gas sparging, medium design, or strain engineering, but these strategies can be expensive, inefficient, or poorly selective. Electro‐fermentation introduces electrodes as electron sinks or donors, allowing external control over redox potential and, in some cases, intracellular NADH/NAD^+^ balance. This can redirect metabolism towards organic acids, alcohols, solvents, chain‐elongated products, biomass, or other biochemicals. Bhagchandanii et al. (2020) describe EF as a way to control redox and pH imbalances, stimulate carbon‐chain elongation or breakdown, and improve product yield through microbe‐electrode interactions. Yamada et al. (2022) sharpen the concept by identifying the key limitation of ordinary fermentation: the net redox equivalents between substrates and products must balance, which restricts the range and efficiency of possible products. EF offers a way to loosen this constraint by using bioelectrochemical systems to modify the intracellular redox state of electrochemically active microorganisms. Recent evidence gives this concept practical weight. Pilania et al. (2025) report that cathodic electro‐fermentation can influence NADH/NAD^+^ balance, improve product titre, reduce byproduct accumulation, and support butyric acid formation from rice straw. Hiebl and Fuchs (2025) showed that even low current densities in electro‐enhanced gas fermentation could reproducibly shift product patterns in *Clostridium carboxidivorans* , *Alkalibaculum bacchi* , and a co‐culture involving *Clostridium kluyveri* , increasing acetate, butyrate, caproate, and alcohol formation under applied potential. The limitation is that EF is not yet a fully predictable industrial tool. Current flow can be low, mechanisms are still not always resolved, and product shifts may depend strongly on cathode surface area, strain physiology, electrode potential, gas composition, and biofilm formation. Nawab et al. (2026) describe this transition well: EF has moved beyond marginal productivity gains in some recent systems, but viable industrial use requires better strain selection, genetic design, scalable biofilm control, and pilot‐scale validation. Thus, electro‐fermentation is best understood as redox process engineering. It does not replace fermentation. It adds an electrochemical control layer that can make fermentation more selective, less aeration‐dependent, and potentially more adaptable to waste and gas feedstocks.

### 8.4 Microbial Electrosynthesis and the Broader Bioelectrosynthesis Context

Microbial electrosynthesis (MES) is used here specifically for electrode‐driven synthesis in which microorganisms or microbial consortia catalyse the conversion of cathodic reducing power and carbon substrates into products. Bioelectrosynthesis is the broader umbrella term and can include other biological electrocatalysts; the two terms are therefore not treated as interchangeable. MES links renewable electricity, carbon conversion, and microbial catalysis under comparatively mild conditions. Sonawane et al. (2021) describe electrode material, biofilm development, system architecture, and electron delivery as core design variables, while Quraishi et al. (2021) position MES and electro‐fermentation as complementary routes for CO_2_ valorisation. Reported product‐formation rates span orders of magnitude; the 3.5–5700 mg L^−1^ d^−1^ range summarised by Sun et al. (2025) pools different products, reactors, cathodes, inocula, operating conditions, and normalisation bases and should therefore be read as evidence of dispersion rather than as a benchmark range. Boto et al. (2025) further show that product scope can be broadened through metabolic and system engineering. The central constraints are electron‐uptake mechanism, rate, selectivity, carbon and electron recovery, product separation, and energy balance. MES is consequently most credible as a targeted platform for CO_2_ or waste‐gas upgrading and specialised chemical production where microbial selectivity provides a clear process advantage.

### 8.5 Industrial Bioprocess Intensification

Industrial intensification should be judged by whether an electrode‐assisted microbial step solves a defined process bottleneck. Scale‐up reviews identify reactor architecture, internal resistance, electrode cost, stability, and long‐term performance as recurring constraints (Corona‐Martínez et al. 2025). Primary cathode studies further show that transport phenomena such as hydroxide movement can be engineered independently of the microbial catalyst and can materially affect system output (Wang et al. 2014). The practical implication is modular integration rather than universal replacement: electroactive microbiomes can be coupled with anaerobic digestion, membranes, advanced oxidation, ion removal, fermentation, or digital control when they improve redox balance, product selectivity, contaminant degradation, methane stability, hydrogen recovery, or dilute‐resource capture. Hybrid electro‐bioreactor concepts illustrate this system‐level direction (Mkilima 2026), while non‐microbial electrochemical hybrids demonstrate that complementary treatment steps may be necessary for salts, metals, or recalcitrant contaminants (Abduova et al. 2025). Industrial value therefore depends on process fit, mass and energy balance, durability, and maintainability under the actual waste stream, not on the highest isolated laboratory current or removal percentage.

## 9 Emerging Translational Boundaries: Host‐Associated and Bioelectronic Systems

This section retains host‐associated and bioelectronic topics only as translational boundaries. Direct evidence is strongest for microbial electrochemical sensing and for selected host‐associated bacteria with experimentally demonstrated EET machinery. Bioelectronic medicine, neural interfaces, smart textiles, and living materials are adjacent frameworks that contribute design principles rather than equivalent evidence of electroactive‐microbiome function. The environmental applications in Sections 7 and 8 therefore remain the principal translational focus, and the subsections below explicitly separate direct microbial electrochemical evidence from analogy or materials‐level extrapolation.

### 9.1 Biosensors and Diagnostic Platforms

**FIGURE 8:**
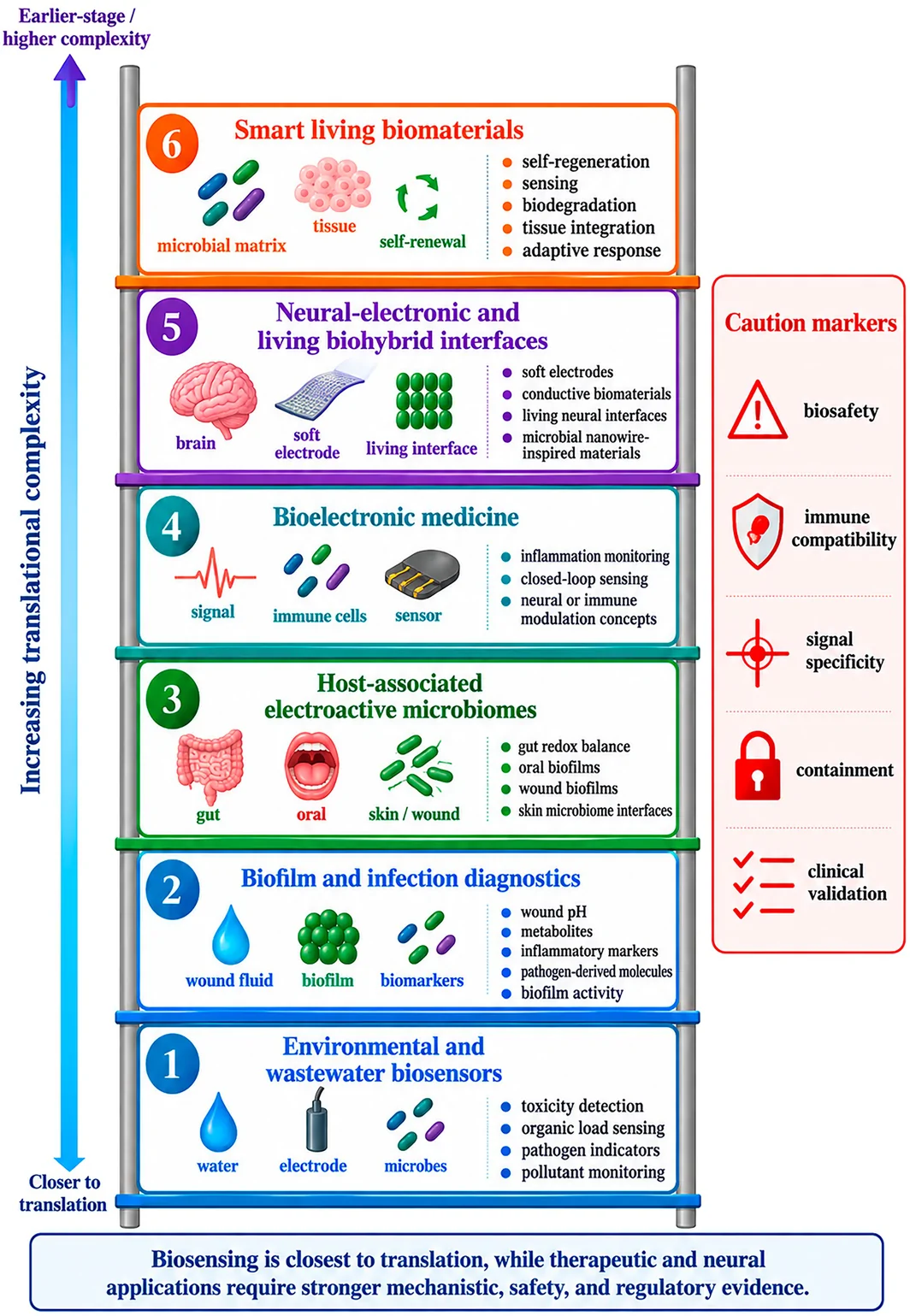
Translational maturity gradient for electroactive‐microbiome and bioelectronic applications.

Microbial electrochemical biosensors provide the strongest translational evidence because electroactive biofilms can convert biologically meaningful perturbations into measurable current, voltage, impedance, or voltammetric responses. In water and wastewater monitoring, altered EET can report toxicity, biodegradable‐substrate changes, metals, or other stressors; Bindu et al. (2024) synthesise this environmental monitoring role, while Chu et al. (2021) show directly that toxicity‐induced changes in electroactive biofilm‐electrode transfer can provide early‐warning signals. Broader water‐quality platforms can add selective recognition layers for emerging contaminants and pathogens (Fdez‐Sanromán et al. 2025), and microfluidic electrochemical systems enable controlled study of biofilm dynamics, virulence, metabolism, and environmental response (Abouhagger et al. 2024). The principal limitation is signal specificity: current loss can also arise from substrate depletion, pH change, detachment, or electrode fouling. These devices are therefore most defensible when calibrated against operating variables and paired with confirmatory electrochemical or molecular measurements. Their near‐term value lies primarily in wastewater surveillance, environmental diagnostics, and biofilm monitoring, with biomedical extensions requiring application‐specific validation (Figure 8).

### 9.2 Host‐Associated Electroactive Microbiomes

Host‐associated electroactivity is a narrower and less resolved evidence domain. Bacterial EET occurs in plant and animal ecosystems and has been linked to fitness, colonisation, redox balancing, and metabolism in organisms such as *Streptococcus mutans* , *Enterococcus faecalis* , *Listeria monocytogenes* , and *Pseudomonas aeruginosa* (Stevens and Marco 2023). Comparative work between corrosion and gut microbiomes highlights shared anoxic niches, biofilm formation, redox cycling, syntrophy, and metabolic adaptability (Jones and El Aidy 2025), but these similarities do not establish direct host‐microbe electron exchange. The better‐supported interpretation is that EET may help particular microorganisms adapt to host redox conditions and influence community metabolism, whereas effects on host physiology are more securely connected through metabolites, inflammation, oxidative stress, and immune signaling (Herrera‐Quintana et al. 2026). Establishing a causal host role for electroactivity will require functional metagenomics or transcriptomics coupled with electrochemical assays, pathway perturbation, and controlled host‐microbe models rather than taxonomic detection alone.

### 9.3 Adjacent Bioelectronic and Living‐Material Frameworks

Bioelectronic medicine demonstrates that physiological regulation can be accessed through controlled electrical interfaces, but it is not itself evidence that microbial systems are established therapeutic bioelectronics. Olofsson and Tracey (2017) and Pavlov et al. (2020) describe neural‐circuit modulation and the inflammatory reflex as a clinical framework for electrically mediated intervention. For electroactive microbiomes, the transferable principle is narrower: a living interface may be electrically monitored or modulated only when signal origin, stability, specificity, and safety are established. Wearable wound sensors already provide a practical adjacent platform for continuous measurement of pH, temperature, inflammatory mediators, metabolites, oxidative stress, and pathogen‐associated signals (Zuo et al. 2025). Electroactive‐microbiome contributions to therapy should therefore remain a future hypothesis until microbial EET is shown to add a controllable and clinically meaningful function beyond conventional sensing.

Neural‐electronic and organic‐bioelectronic research contributes an interface‐design analogy rather than a demonstrated microbial neural technology. Soft, biohybrid, and living neural interfaces are being developed to reduce mechanical mismatch, inflammation, and unstable tissue‐electrode contact (Boufidis et al. 2025), while living‐biomaterial neuroelectronics seek adaptive bidirectional coupling (Hong et al. 2026). Organic bioelectronics further shows how mixed ionic‐electronic conductors can bridge ion‐dominated biological signalling with electronic devices (Moiz et al. 2025). Microbial nanowires and conductive living structures may eventually contribute materials to this design space, but direct neural recording or stimulation by microbial biofilms remains preliminary. Relevant translation criteria are biocompatibility, purity, electrical stability, controllability, and long‐duration functional testing.

Living‐material research provides a second adjacent framework with a stronger direct connection to electroactive microbial structures. Electroactive bacteria and microbial nanowires are being explored as biodegradable electronic materials, including cable‐bacteria‐derived conductive structures (Bonné et al. 2022); engineered living energy materials add self‐growth, regeneration, biodegradability, and environmental responsiveness (Yuan, Xu, et al. 2025); and microbiome‐electronics concepts extend these principles to smart textiles, e‐skin, wound dressings, and microbe‐integrated devices (Wu et al. 2026). These advantages are inseparable from containment challenges: living components require water and nutrients, can change function over time, and may interact with host or environmental microbiota. Accordingly, this area is best treated as a materials frontier whose progression depends on stable conductivity, controlled activation and shutdown, biosafety, immune compatibility where relevant, and reproducible device performance.

## 10 Challenges, Risks, Limitations and Future Frontiers

Environmental deployment is now limited less by proof‐of‐principle than by predictable performance under realistic feed variability, long operation, changing environmental conditions, maintenance constraints, cost, safety, and governance. Laboratory demonstrations establish that electroactive communities can exchange electrons with electrodes, transform contaminants, and recover products, but they do not by themselves establish field reliability. The remaining challenges can be separated into reactor and biofilm bottlenecks, standardisation and reproducibility, techno‐economic and life‐cycle performance, biosafety and regulation, and the ability to control community function over time. Treating these constraints explicitly is essential for distinguishing scalable environmental biotechnology from short‐duration laboratory optimisation.

### 10.1 Technical and Operational Limitations

The persistent technical limitation is the gap between cell‐ or biofilm‐scale EET and whole‐reactor performance. Mass‐transfer resistance, proton accumulation, substrate gradients, internal resistance, electrode passivation, membrane fouling, cathodic losses, biofilm overgrowth, detachment, and reactor geometry can all suppress system output. Netsch et al. (2025) reported maximum current at mean mixed‐community biofilm thicknesses of approximately 100–150 μm in their microbial electrolysis configuration, with thicker biofilms developing mass‐transfer limitations and local detachment. This does not conflict with the approximately 20 μm optimum discussed earlier for a *Geobacter* system; rather, the difference demonstrates that biofilm optima are system‐specific and depend on community composition, substrate supply, electrode geometry, hydrodynamics, buffer capacity, and current demand. Broader bioelectrochemical evidence likewise identifies electrode selection, device design, microorganism selection, and electron‐transfer architecture as coupled determinants (Zheng et al. 2020). During scale‐up, real wastewater adds solids, inhibitors, salinity, temperature shifts, competing guilds, and maintenance requirements that are largely absent from defined laboratory feeds. Circular‐bioprocess studies further show that product recovery and microbial kinetics can dominate economics even when biological conversion is feasible (Tong et al. 2023). Reactor development should therefore optimise stable transport and electron recovery over time, not peak short‐term current density alone.

### 10.2 Standardisation, Biosafety, Economic and Regulatory Barriers

The translational barriers facing electroactive microbiome technologies are not only engineering problems. They also include weak standardisation, inconsistent reporting, uncertain biosafety, limited techno‐economic evidence, regulatory ambiguity, and poor reproducibility across laboratories. Many studies report power density, current density, coulombic efficiency, COD removal, product titre, or removal efficiency, but these metrics are often measured under different reactor volumes, electrode areas, substrates, inocula, operating times, and normalisation methods. This makes cross‐study comparison difficult and can exaggerate apparent progress. Similar problems are visible across broader microbiome research. Filardo et al. (2024) emphasise that microbiome profiling has advanced through 16S rRNA sequencing, whole‐genome sequencing, transcriptomics, metabolomics, and proteomics, but translation remains limited by methodological variability, incomplete functional interpretation, and the need for stronger multi‐omics integration. Fadiji et al. (2025), writing on plant microbiome research, identify standardisation, reproducibility, metadata quality, environmental variability, host specificity, biosafety, ethical concerns, regulatory pathways, and economic viability as core barriers to field‐scale use. These issues apply directly to electroactive microbiomes because their function depends on community composition, electrode material, redox environment, reactor design, and environmental context. Biosafety adds another layer. Engineered electroactive consortia, bioaugmentation strategies, conductive materials, and synthetic biology tools may improve performance, but they also raise concerns about ecological release, horizontal gene transfer, antimicrobial resistance genes, and long‐term persistence outside controlled reactors. Renganathan et al. (2025) highlight these risks in engineered microbial consortia for xenobiotic wastewater treatment, especially microbial instability, antibiotic resistance gene dissemination, reactor fouling, and the absence of region‐specific microbial reference databases. Economic feasibility is equally decisive. A system that removes pollutants but requires expensive electrodes, fragile membranes, high maintenance, or complex control will struggle against established treatment technologies. For electroactive microbiomes, commercialisation will depend less on demonstrating novelty and more on showing verified advantages under standardised operating conditions, with life‐cycle assessment, techno‐economic analysis, biosafety assessment, and regulatory‐ready performance data. The field, therefore, needs shared reporting frameworks, benchmark substrates, reference inocula or community descriptors, minimum operation durations, standardised electrode‐area normalisation, clear product recovery metrics, and transparent accounting of energy inputs and material costs. Without these, promising platforms will remain difficult to compare, regulate, finance, or deploy.

### 10.3 Future Frontiers and Next‐Generation Electroactive‐Microbiome Platforms

Electroactive‐microbiome research is beginning to generate datasets large enough for system‐specific machine learning, but the strongest current role is decision support. Park and He (2026) integrated 136 observations from 44 photosynthetic microbial‐fuel‐cell studies and used interpretable machine learning to identify multivariate drivers of COD removal and power generation despite heterogeneous reporting. Yoon et al. (2024) constructed random‐forest models from 76 microbial‐electrolysis‐cell datasets to predict current and hydrogen production and showed both the predictive value of applied voltage and cathode area and the difficulty of encoding categorical variables such as electrode material. These studies demonstrate useful cross‐study pattern extraction; they do not resolve mechanistic uncertainty. Future models should therefore be trained on standardised electrochemical, microbial, material, hydraulic, and long‐duration operational descriptors and validated outside the data used for fitting. Community engineering provides a complementary route. Foo et al. (2017) describe microbiome engineering as deliberate modification of community composition or function, but the framework is broader than electroactive systems and must be adapted cautiously to electrode‐associated ecology. A credible design‐build‐test‐learn cycle would combine multi‐omics to identify active functions, mechanistic or metabolic models to locate constraints, ML to prioritise interventions, selective enrichment or synthetic biology to modify defined targets, and real‐time electrochemical sensing to test whether electron flux and process performance improve. Autonomous control, designed consortia, and living biohybrid interfaces remain emerging directions until long‐term stability, containment, reproducibility, and environmental benefit are demonstrated.

## 11 Conclusion

Electroactive microbiomes provide a mechanistically distinct framework for environmental microbiology in which extracellular electron flow links community metabolism to minerals, pollutants, partner organisms, and engineered electrodes. Across the evidence synthesised here, the most mature applications are wastewater treatment and resource recovery, anaerobic‐digestion support, pollutant and metal transformation, and microbial electrochemical recovery of electricity or reduced products. Soil and sediment engineering and carbon conversion have growing experimental support but need more long‐duration, field‐relevant validation, while host‐associated and bioelectronic concepts remain emerging translational boundaries. Translation depends not on maximising a single current or removal metric but on reproducible performance under realistic feed variability, standardised reporting, long‐duration operation, defensible mechanistic attribution, energy and mass balances, techno‐economic and life‐cycle assessment, biosafety, and regulatory compatibility. Mechanistic studies should resolve pathway‐specific electron flux in mixed communities and distinguish direct, mediated, interspecies, and material‐assisted transfer. Engineering studies should couple biofilm and electrode design with mass and charge transport and community stability. Multi‐omics, metabolic modelling, and artificial intelligence are most useful when experimentally constrained and used as decision‐support tools. These priorities position electroactive microbiomes primarily as environmental microbial systems for circular treatment, resource recovery, carbon routing, and redox management, while keeping biomedical and bioelectronic extensions explicitly proportional to the available evidence.

## Author Contributions

**Timoth Mkilima:** conceptualization, writing – original draft, writing – review and editing.

## Conflicts of Interest

The author declares no conflicts of interest.

## Data Availability

Data sharing not applicable to this article as no datasets were generated or analysed during the current study.
